# GrandQC adaptation as an artificial intelligence tool for quantitative artifact detection in hematoxylin and eosin whole-slide images—Simulation of quality control biopsies day

**DOI:** 10.1016/j.jpi.2026.100682

**Published:** 2026-06-11

**Authors:** Gonçalo Borrecho, Pedro Nina, Ricardo Santos, Inês Ferreira, Pedro Salgueiro, Catarina Madeira, Luís Rato, Rui Caetano Oliveira

**Affiliations:** a NOVA National School of Public Health, NOVA University Lisbon, Lisbon, Portugal; b Comprehensive Health Research Centre (CHRC), NOVA Medical School, Lisbon, Portugal; c Pathology Department, Unidade Local de Saúde de Coimbra (CHUC), Coimbra, Portugal; d CoLAB TRIALS Association, Évora, Portugal; e Egas Moniz Center for Interdisciplinary Research (CiiEM), Monte de Caparica, Portugal; f Human Genetic Department, Instituto Nacional de Saúde Doutor Ricardo Jorge (INSA), Lisbon, Portugal; g Nova Information Management School (IMS), Lisbon, Portugal; h Centro de Anatomia Patológica Germano de Sousa (CAPGS), Coimbra, Portugal; i VISTA Lab, Centro Algoritmi, Universidade de Évora (UE), Évora, Portugal

**Keywords:** Digital pathology, Computational pathology, Artificial intelligence, Quality control

## Abstract

**Introduction:**

Digital pathology continues to transform the daily routine of pathology, in terms of the increasingly automated laboratory and in the diagnostic paradigm through the adoption of artificial intelligence (AI) tools to support diagnosis—computational pathology. The reliability and performance of these tools depend on the whole-slide image (WSI) quality being guaranteed a priori. Pre-analytical quality control step that underpins this guarantee, and artifact detection remains largely qualitative and is frequently overlooked in routine digital pathology. This operational feasibility study evaluated whether an adaptation of GrandQC, an open-source AI tool, enables automated, quantitative artifact assessment of a complete single-day biopsy workload from a high-throughput digital pathology laboratory, analyzed retrospectively.

**Material and methods:**

A random biopsies day of 2025 at Centro de Anatomia Patológica Germano de Sousa (CAPGS) was selected as a sample to test the performance of GrandQC on the WSI generated (*n* = 544) in order to simulate the daily workflow. A script was created to quantify the pixels corresponding to the type of artifact automatically, creating an Excel file for registering and statistical analysis.

**Results:**

Analysis took a median of 24 s per WSI, detecting a median of 1.46% of tissue area with some type of artifact. Dark Spots and blurring areas were the most representative detected artifacts.

**Conclusion:**

GrandQC is a valuable tool in the quantitative quality control of biopsies tissue, allowing quick evaluation, signaling types of artifacts, and identifying cases that need to be reviewed before being handed over to the pathologist allowing the recognition of opportunities to improve laboratory histology quality and precision medicine.

## Introduction

Digital pathology represents a profound transformation in pathological anatomy lab, enabling the transition from conventional microscopes to the computational analysis of digitized histological slides—whole-slide images (WSI).^1^, ^2^, ^3^, ^4^, ^5^, ^6^ At the heart of this revolution is deep learning (DL), an advanced artificial intelligence (AI) technology that uses neural networks to analyze these digital images. DL systems have demonstrated their ability to achieve remarkable accuracy, rivaling that of human pathologists in tasks such as prostate cancer detection, where accuracy can exceed 98%,^7^ in support in routine, laborious, and repetitive situations such as the determination of the grade of infection by *Helicobacter pylori*, mitosis scoring in breast cancer, as well as in the determination of predictive biomarkers without the use of complementary diagnostic techniques such as HER2, microsatellite instability, and EGFR mutations, despite these resources only being available for research.^8^, ^9^, ^10^, ^11^, ^12^ This change enables the integration of DL algorithms for routine tasks, such as tumor detection and grading, promising to objectify, quantify, and personalize cancer diagnosis, removing subjective variability.^13^, ^14^

However, despite this enormous potential, the transition of DL models from the lab to the real world is complex. Their accuracy faces a major challenge: AI algorithms, which depend on consistent mathematical patterns, face the reality of pathology, which is inherently variable and imperfect. In other words, the clinical implementation of these tools faces a critical obstacle: the high heterogeneity of slides and the ubiquitous presence of artifacts.^15^, ^16^

Artifacts are imperfections that arise in histological slides during the various stages of histological processing, from initial tissue processing to final digitization. For an AI model, an artifact is not just “dust” or a “fold,” it is a new set of unexpected visual data that do not match the patterns it has learned, and may corrupt its analysis or be interpreted as a false pathological feature.^17^, ^18^, ^19^, ^20^, ^21^

Scientific evidence suggests that any of the artifacts studied, depending on their severity, can result in a significant loss in the performance of an AI model, constituting a significant “bottleneck” not only for computational pathology but also for diagnosis per se.^21^, ^22^, ^23^, ^24^ Notably worrisome is the fact that AI models often fail “silently,” incorrectly classifying areas with artifacts (such as tissue folds or out-of-focus) without issuing warnings of uncertainty. Out-of-focus is among the most critical artifacts: Schömig-Markiefka et al. (2021) demonstrated that blurring still visually tolerable to a pathologist can cause unacceptable accuracy losses for an algorithm, with a level-2 blur—an intermediate grade on the graded blurring scale defined in that study, corresponding to a moderate, still visually subtle loss of focus—misclassifying 4.95% of tumors as benign (false negatives)—the most dangerous error in a clinical context, as it represents a “silent failure” that, unlike a false positive, goes uncorrected. Conversely, removing artifact-affected image patches has been shown to improve model performance, with reported AUC gains from 0.88 to 0.92–0.93 in metastasis detection and lung cancer classification, confirming that rigorous QC is a prerequisite for reliable computational pathology.^23^, ^24^

Thus, in a situation where thousands of histological slides may be produced for analysis, however small the percentage of error, it is important to detect these flaws/artifacts early, in an easy, direct, and reliable manner that has the least possible impact on turnaround times.^25^, ^26^, ^27^, ^28^, ^29^ This activity is a technical responsibility, which, when performed manually, is subjective, laborious, qualitative, and subject to potential intra- and inter-observer bias.^22^, ^27^, ^30^, ^31^, ^32^ However, based on the idea that this type of artifact occurs in less than 5% of scanned slides, in the context of a validated digital pathology, it is not cost-effective to allocate scarce human resources for quality control purposes, as it is sufficient to request a repeat.^1^, ^22^, ^25^, ^33^, ^34^ One study suggests the need for one full-time employee for every three to four high-volume scanners, an unsustainable resource requirement for most labs.^35^ This reliance on subjective and error-prone human verification represents a weak link in the digital pathology workflow. Reported digitization error rates are highly heterogeneous, ranging from 0.27 to 29.5%, and for 45% of scanner-equipped labs in Europe and Asia they represent the primary impediment to the full adoption of digital pathology.^19^, ^33^, ^34^, ^36^, ^37^, ^38^, ^39^, ^40^, ^41^, ^42^ Hence, the lab is constrained between the guarantee of maintaining technical rigor and managing scarce human resources.

To mitigate these operational risks, the transition to systematic, objective, and automated quality control supported by AI tools becomes necessary—a process in which the histotechnologist plays a central role as the guarantor of slide and WSI quality.^25^, ^43^, ^44^

**Fig. 1:**
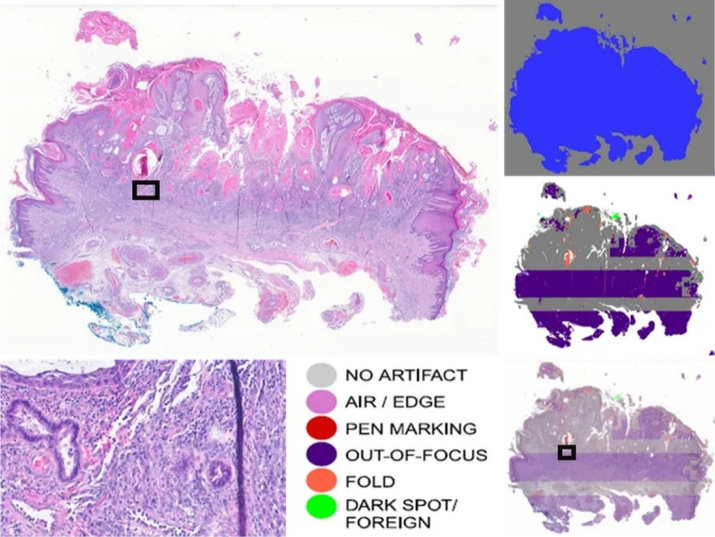
Example of GrandQC's detection of tissue and its artifacts (density map), showing areas of blur/out of focus (purple) stitching error and areas of folded tissue (orange). The color legend is adapted from Weng et al. (2024). (For interpretation of the references to color in this figure legend, the reader is referred to the web version of this article.)

The GrandQC tool emerges as a comprehensive solution to this problem. It is an open-source deep-learning framework built on a modular architecture of two pixel-level segmentation networks—one for tissue detection and one for multi-class artifact detection—covering the most frequent and disruptive artifacts in histological slides, including air bubbles, slide edges, blurred areas, pen marks, tissue folds, foreign bodies, and dark spots (Fig. 1). The tool was developed and validated on a large, manually annotated dataset drawn from 19 international pathology centers and The Cancer Genome Atlas, across multiple scanners and platforms, and has demonstrated accuracy superior to other available quality-control tools while analyzing a slide in less than a minute. This extensive, multi-institutional validation already establishes the technical reliability and cross-institutional generalizability of the tool; the present work, therefore, does not seek to re-validate its detection performance, but to evaluate its operational feasibility when applied, externally and independently, to the routine biopsy workflow of a high-throughput lab.^22^, ^24^, ^45^, ^46^, ^47^

The application of GrandQC in a real daily lab workflow of biopsies, externally and independently, is important and rarely described in the literature. The importance of this challenge extends beyond image quality itself. Because distinct artifact types map onto distinct stages of histological processing, their systematic and quantitative monitoring shifts quality control from a passive, qualitative metric towards an objective basis for lab quality management, the early detection of process deviations, and compliance with certification and accreditation standards—making its systematic study a priority for digital pathology.^20^, ^25^, ^27^, ^45^, ^48^, ^49^, ^50^

This operational feasibility study evaluated whether an adaptation of GrandQC enables automated, quantitative artifact-based quality control of WSIs within a routine high-throughput biopsy workflow, analyzed retrospectively. The specific objectives were: to assess the operational feasibility of the adapted GrandQC pipeline for quantitative artifact detection across a complete single-day biopsy workload; to quantify the prevalence, distribution, and magnitude of each artifact type; and to characterize the relationship between artifact burden and operational parameters, namely analysis time, scanning time, and file size.

## Methods

### Investigation site

The Centro de Anatomia Patológica Germano de Sousa (CAPGS) is a large private pathological diagnosis center located in Coimbra, Portugal, with an area of influence that effectively extends throughout the country. Every year, the lab processes around 180,000 samples, of which approximately 110,000 correspond to cases of histopathology and other diagnostic fields, including cytopathology, fetopathology, and molecular pathology.

CAPGS's digital pathology program was launched in 2020 with the dual goal of supporting routine primary diagnosis and improving multi-disciplinary case review. The lab currently operates three high-throughput Aperio GT450DX (Leica Biosystems) scanners, which generate images of entire slides at 40× magnification (0.26 μm/pixel), and uses the Aperio eSlide Manager WebViewer as a viewing platform for case evaluation. The digital environment is closely linked to the existing lab information system (SISPAT) through Health Level 7 (HL7) messages, enabling fully integrated digital workflows and continuous case tracking, from access to report authorization.

A comprehensive re-engineering of processes based on LEAN underpinned the transition, enabling better use of available physical space and the incorporation of digital steps without increasing the number of technicians. The lab operates under a quality management structure certified according to ISO 9001 requirements, which provided an established framework for monitoring and improving digital implementation. Special emphasis was placed on validating and ensuring the quality of digital diagnosis, in accordance with the recommendations of the Royal College of Pathologists, as detailed in the validation study previously reported by Ferreira et al. (2023).^34^

The CAPGS digital ecosystem is subject to continuous improvement, with progressive integration and updating of instruments and systems using standardized communication protocols. Ongoing quality initiatives include systematic documentation of errors, robust traceability through combined digital and physical archiving solutions, and continuous training of technical and medical staff. Within this framework, the lab has successfully migrated to fully remote assignment of histological cases to subspecialist pathologists, ensuring flexibility and maintaining diagnostic capacity.

Daily, the samples are confirmed, received, and registered, where they are assigned a sequential internal case number, generating a data matrix code, which is scanned in the macroscopic analysis, from the container and/or request and used to print the corresponding cassette. Biopsies are fixed in 4% buffered formaldehyde, dehydrated, diaphanized, and impregnated in paraffin, undergoing histological processing overnight using the standard biopsy protocol of the LEICA PELORIS® processor. The biopsies are then embedded and cut individually to a thickness of 3 μm, passing through the monitoring/tracking system (Lab information system, LIS) to avoid errors and ensure quality. H&E staining and glass coverslip mounting are performed automatically by LEICA SPECTRA® equipment, using a validated protocol. As this process is completed, the slides are individually checked for quality standards before scanning, such as slide drying, mounting defects, and staining appearance, moving the slides to a dry and clean support, avoiding the risk of sticking of slides coming from the mounting oven. Only then are they placed in the Aperio GT450DX® scanners with priority scanning, being automatically available to pathologists for analysis/diagnosis, leaving it to their discretion to request a repeat scan if any non-conformity is found. This practice is adopted based on the previous validation process, which found a repetition rate of less than 0.6%.^34^ Therefore, it is very difficult to allocate a scarce technical element to a tedious task, where they would have to continuously check more than 1000 WSIs per day, with the same performance, where intra- and inter-observer bias constitutes a significant risk.^1^, ^2^, ^34^

Although many artifacts observed on glass slides are generally considered to be of limited relevance for diagnostic interpretation, this assumption is not always true in the digital environment, where image quality issues can more directly affect visualization, and even less so in the context of computational pathology. At present, the lab does not have AI algorithms to support diagnosis for use in daily routine, however, the strong possibility of their consideration for future adoption is recognized. Consequently, pre-scanning quality control becomes a critical step in the workflow. However, the shortage of specialized human resources makes it difficult to assign experienced technicians exclusively to quality control tasks, which are repetitive, time-consuming, and susceptible to observer bias. In this context, the implementation of an open-source tool based on AI has the potential to provide substantial support, helping to standardize and optimize quality control in high-throughput digital pathology labs.

### Study setting

A random day of 2025 at CAPGS of biopsies was selected as a sample to test the performance of the GrandQC+ algorithm on the WSI generated in order to simulate the daily workflow. On the selected day, 544 WSIs were obtained from biopsies of different tissue origins, mainly including skin, gastrointestinal tract, lung, breast, prostate, and bladder biopsies, among others, scanned at 40× magnification (0.26 μm/pixel) in one Aperio GT450DX scanner.

Biopsy specimens were deliberately chosen as the focus of this study. Because they are small, often unique and irreplaceable, biopsies are the primary means of detecting diseases and making therapeutic decisions, a context in which image quality is crucial and it is often not possible to collect new samples—unlike with resection specimens from surgical specimens, where samples are taken from suspicious areas to be examined. Their characteristically smaller file size also made it feasible to process a complete single-day workload within the scope of this feasibility evaluation. The biopsy category is itself heterogeneous in size, encompassing excisional biopsies that may approach the dimensions of small resection specimens.

These WSIs obtained in .SVS format were anonymized with WSIANON methodology, described by Bisson et al. in their article, with the purpose of protecting eventual WSI sensible data, and in compliance with the ethical principles and data protection law previously described and defined.^54^

GrandQC was previously installed via the GitHub platform following the suggested instructions on a laptop equipped with a 13th-generation Intel Core i9-13980HX CPU, NVIDIA GeForce RTX 4090 GPU, and 64 GB RAM. Both GrandQC modules are pixel-level semantic segmentation networks, implemented in PyTorch, with an EfficientNetB0 encoder and a UNet++ decoder, each requiring approximately 1.5 GB of GPU memory. The tissue and artifact detection models were deployed exactly as publicly released by the original developers, using the pre-trained checkpoints obtained from the official repository, without local fine-tuning or retraining on CAPGS data.

Analysis followed the standard GrandQC pipeline, in which the two modules operate sequentially: tissue detection at 1× magnification, followed by artifact detection within the segmented tissue regions using the 7× model, the version recommended by the developers for routine use, which balances segmentation accuracy and processing speed. The complete set of 544 WSIs from the selected production day was processed as a single overnight batch on this local, offline workstation, without integration into the LIS. The generated segmentation masks were analyzed empirically and qualitatively to confirm the correct functioning of the algorithm, establishing a visual correspondence between the segmentation mask and the original image.

**Fig. 2:**
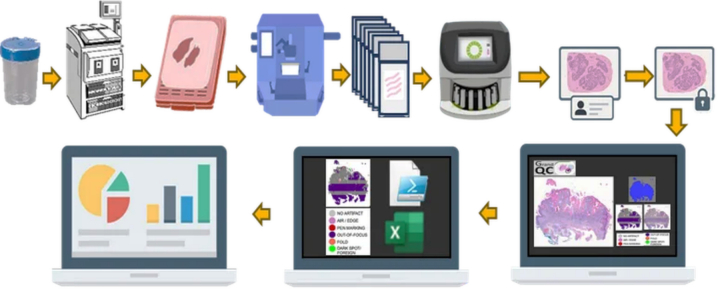
Methodology scheme of a single day of biopsies processed at the CAPGS lab, in which 544 slides were digitised, anonymised with WSIANON and analysed overnight by GrandQC. The developed script analyzed the number of pixels of each color corresponding to the type of each artifact and generated an excel file for statistical analysis.

The native output of GrandQC consists of tissue and artifact segmentation masks accompanied by a text file, which does not by itself allow the proportion of each artifact type to be quantified. To address this, a custom script was developed: a PowerShell wrapper that orchestrates execution across all WSI masks in a directory and invokes a Python routine—based on OpenCV (cv2) and NumPy—which, for each mask, counts the number of pixels matching the RGB code assigned by GrandQC to each segmentation class. Seven classes are quantified: artifact-free tissue (gray, RGB 128,128,128), folded tissue (orange, RGB 255,99,71), dark spots (green, RGB 0,255,0), pen markings/other artifacts (red, RGB 255,0,0), air/edge (pink, RGB 255,0,255), and out-of-focus (violet, RGB 75,0,130), together with the background class (white, RGB 255,255,255). For each WSI, the per-class pixel counts are written into a structured Excel file, together with derived metrics—the percentage of each artifact class relative to total tissue, the total artifact-affected area, the area of artifact-free tissue, the analysis time, the digitization time, the GrandQC+ run time, and the file size—enabling downstream statistical analysis. The script is available from the corresponding author upon reasonable request. This combined framework of GrandQC and the custom quantification script is hereafter referred to as GrandQC+ (Fig. 2).

### Statistical analysis

Descriptive and inferential statistics were performed using Python (version 3.1) with the SciPy and Statsmodels libraries. Descriptive statistics included mean, median, standard deviation, interquartile range (IQR), and percentiles. Normality was assessed using the Shapiro–Wilk and D'Agostino–Pearson tests, both of which rejected the null hypothesis of a normal distribution for all variables under analysis (*p* < 0.001); non-parametric methods were therefore adopted for all inferential analyses. Group comparisons were performed using the Mann–Whitney *U* test and correlations between continuous variables using Spearman's rank correlation coefficient (ρ). Effect sizes were reported for all comparisons, calculated as *r* = *Z*/√*N* for the Mann–Whitney *U* tests, with Spearman's ρ serving as the effect size measure for correlations. Confidence intervals (95%) for medians were estimated by bootstrap resampling (10,000 iterations, with a fixed random seed for reproducibility). Multivariate linear regression was used to evaluate the contribution of technical parameters to artifact load variance. Given the number of pairwise comparisons and correlations performed (23 tests), the Benjamini–Hochberg procedure was applied to control the false-discovery rate across the full set of tests; this approach was preferred over the more conservative Bonferroni correction as more appropriate for an exploratory correlational analysis involving multiple inter-related variables.

The severity grading of WSI artifacts in this study was quantified as the proportion of tissue area affected. In the present dataset, slides were classified as perfect (<1% artifact-affected tissue), good (1%–5%), moderate (5%–10%), or severe (>10%), using thresholds derived from local expert consensus and internal validation to reflect the extent of artifactual involvement considered compatible with reliable diagnostic interpretation. Furthermore, this classification was performed using a data-based threshold, based on statistical detection of outliers, according to Tukey's method, with this threshold defined as Q3 + 1.5 × IQR. Ultimately, WSIs with artifact load ≤10% were classified as minimally acceptable quality, whereas those exceeding this threshold were classified as severe, consistent with what is reported in the literature.^45^, ^55^ These severity thresholds are institution-specific and exploratory in nature, derived from the artifact-load distribution of the present cohort; they are intended as a practical framework for internal quality monitoring rather than as universally validated cut-offs, and labs in different settings should expect to adapt them to their own case mix and digitization conditions.

### Ethics

All ethical principles applicable to the conduct of this study were strictly observed, ensuring respect for the principles of autonomy, beneficence, non-maleficence, and justice, in accordance with the Oviedo Convention and in strict compliance with data protection legislation enshrined in the General Data Protection Regulation (GDPR).^51^, ^52^ In line with the principle of data minimization, no personal data were processed or analyzed at any stage of the work.^53^ The principal investigator did not, at any time, have access to personal data or the identification of patients relating to the samples included in the study. All experimental activity carried out at CAPGS was anonymized using the open-source WSIANON methodology, in accordance with the GDPR, making it impossible to identify or re-identify patients.^54^ Furthermore, this is a retrospective study, conducted as an institutional quality improvement initiative, which does not fall within the scope of a clinical study and does not involve any type of intervention, analysis, or processing of personal data. The study was conducted with the documented authorization of the CAPGS lab management and, in accordance with the applicable Portuguese regulatory framework and institutional policy, retrospective quality improvement studies of this nature are exempt from formal review by an ethics committee; consequently, no ethics committee approval number is applicable.

## Results

A total of 544 slides were successfully scanned, with the scanning process completed in 7 h and 10 min (48 s on average per WSI), generating a total of 328 gigabytes (GB) of WSI data, with a mean and median of 0.6 and 0.34 GB (95% CI: 0.30–0.39), respectively. No technical errors or failures were detected during the scanning procedure.

The subsequent step of anonymizing the complete set of WSIs was completed in approximately 10 min without any issues.

Automated image quality assessment with the GrandQC+ tool was performed on all WSIs, requiring a total processing time of 8 h and 39 min. This corresponds to a mean analysis time of 57 s per WSI, with a median of 24 s (95% CI: 24–30), indicating that a substantial proportion of cases were processed faster than the average value. The execution of GrandQC+ resulted in a total of 1.45 GB of segmentation mask files, which summarize the spatial distribution of artifacts across the image set.

The segmentation of the WSIs proceeded without intercurrences, correctly identifying the tissue area to be analyzed for artifact detection through visualization and empirical analysis of the generated masks.

**Fig. 3:**
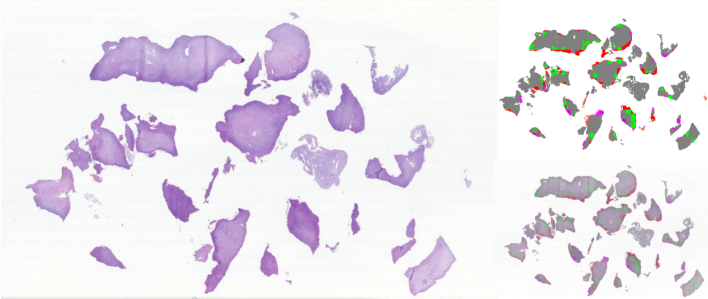
WSI of transurethral resection of the prostate (TURP) with peripherical areas of cauterization interpreted by the GrandQC as “pen markings” (in red) posteriorly designated by “other artifacts.” (For interpretation of the references to color in this figure legend, the reader is referred to the web version of this article.)

With regard to artifacts, applying the newly developed script, 97.1% (95% CI: 95.3%–98.2%) have some type of artifact (>0%), identifying 16 WSI completely free of artifacts (0 pixels corresponding to some type of artifact). The most prevalent artifact present in the sampling were dark spots identified in 514 WSI (94.5%), followed by pen markings present in 454 WSI (83.5%), folded tissue in 322 (59.2%), air/edge in 321 (59%), and out-of-focus in 220 (40.4%). It should be noted that the digitized sample did not have any pen markings, as it is the production of original slides before diagnosis and before assignment to the pathologist, however, GrandQC+ reported positive detections in 83.5% of the cases (*n* = 454). The percentage of tissue area affected by this artifact were 0.48% and 0.06% of average and median, respectively, making its detection almost irrelevant. Despite this, a new nomenclature was assigned to this type of detected artifact, now called “other artifacts” (Fig. 3).

**Fig. 4:**
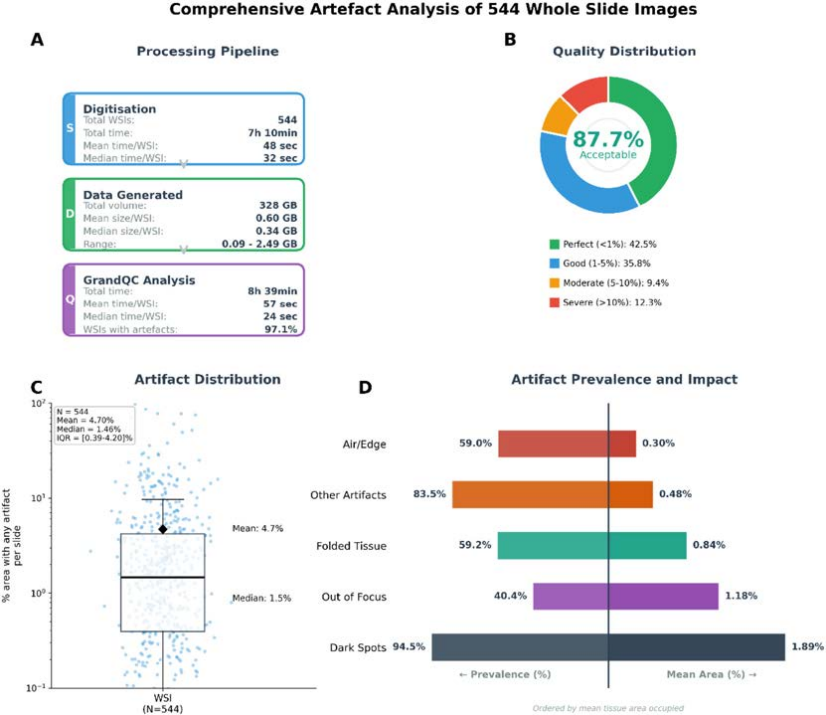
Descriptive statistics of the results obtained.

The percentage of affected tissue pixels in WSIs had a mean value of 4.7%, with a median of 1.46% (IQR: 0.39%–4.20%; 95% CI: 1.23%–1.73%), suggesting that most slides showed a relatively low artifact load. The most representative type of artifacts were dark spots (1.89%) and blurred areas (1.18%), which were the main quality issues identified in the scanned images. Within this group, only one WSI required repetition of the digitization process due to a stitching error during image assembly. Other artifacts and air/edge were the artifacts with the lowest percentage of tissue area occupied (mean of 0.48% and 0.30%, respectively; Fig. 4).

Categorization by severity showed that 87.7% of WSIs were of satisfactory quality—42.5% perfect (≤1% of tissue area occupied by some type of artifact), 35.8% good (>1%–≤5%) and 9.4% moderate (>5%–≤10%), whereas 12.3% were of high severity (≥10%).

**Fig. 5:**
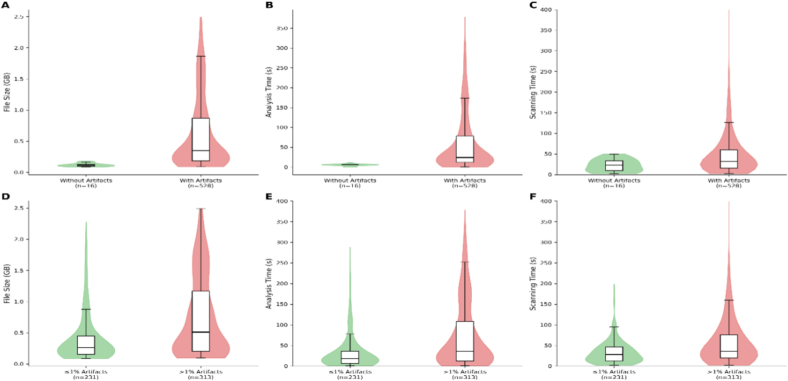
Comparison of differences between groups “with and without artifacts” and ≤1% and >1% artifacts in relation to performance variables (A and D—file size; B and E—analysis time; C and F—scanning time).

The comparison between WSIs with detectable artifacts (*n* = 528) and WSIs without artifacts (*n* = 16) was conducted using the Mann–Whitney *U* test, revealing statistically significant differences in all evaluated technical parameters. The file size differed between the groups, with median values of 0.35 GB (IQR: 0.19–0.87) in WSIs with artifacts versus 0.11 GB (IQR: 0.10–0.13) in WSIs without artifacts (*p* < 0.001, *r* = 0.252). The GrandQC+ analysis time showed a median of 24.00 s (IQR: 12.00–78.00) for WSIs with artifacts and 6.00 s (IQR: 6.00–6.00) for WSIs without artifacts (*p* < 0.001, *r* = 0.237). The scanning time was also higher in WSIs with artifacts (median: 32.00 s; IQR: 16.00–60.00) compared to WSIs without artifacts (median: 23.00 s; IQR: 9.50–33.50), with *p* = 0.019 and *r* = 0.101 (Fig. 5A–C).

Were also considered for comparison, using the same methodology, between WSIs with minimal artifact load (≤1% of tissue area; *n* = 231) and WSIs with higher artifact load (>1%; *n* = 313). The file size also differed between the groups, with median values of 0.51 GB (IQR: 0.20–1.17) in WSIs with >1% artifacts versus 0.26 GB (IQR: 0.15–0.45) in WSIs with ≤1% artifacts (*p* < 0.001, *r* = 0.277). The GrandQC+ analysis time showed a median of 36.00 s (IQR: 12.00–108.00) for WSIs with >1% artifacts and 18.00 s (IQR: 6.00–36.00) for WSIs with ≤1% artifacts (p < 0.001, *r* = 0.271). The scanning time showed a median of 36.00 s (IQR: 20.00–76.00) for WSIs with >1% artifacts and 28.00 s (IQR: 13.00–46.00) for WSIs with ≤1% artifacts (*p* < 0.001, *r* = 0.215; Fig. 5D–F). Interestingly, whereas statistically significant differences were observed between WSIs with ≤1% and >1% artifact load across all technical parameters, no significant differences were found when comparing acceptable quality WSIs (≤10% artifacts; *n* = 477) with severe cases (>10%; *n* = 67). In this latter comparison, tissue coverage (median: 9.03% vs 8.75%; *p* = 0.594), file size (median: 0.34 GB vs 0.37 GB; *p* = 0.452), GrandQC+ analysis time (median: 24.00 s vs 30.00 s; *p* = 0.350), and scanning time (median: 32.00 s vs 36.00 s; *p* = 0.070) showed no statistically significant differences, with negligible effect sizes (*r* < 0.1) for all parameters.

Regarding the correlations between artifacts and technical parameters, the total number of artifacts showed very weak positive correlations with tissue coverage (ρ = +0.12, *p* < 0.01), file size (ρ = +0.26, *p* < 0.001), analysis time (ρ = +0.25, *p* < 0.001), and scanning time (ρ = +0.19, *p* < 0.001), reflecting a modest impact of artifact load on performance. The strongest correlation was observed between the air/edge artifact and the analysis time (ρ = +0.56, *p* < 0.001), whereas the weakest correlation was found between the total number of artifacts and tissue coverage (ρ = +0.12, *p* < 0.01).

**Fig. 6:**
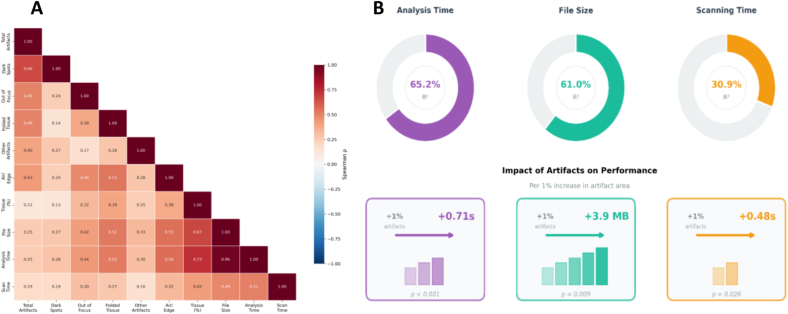
(A) Spearman correlations between variables. (B) Multivariate regression showing artifact determinants and operational impact per 1% artifact increase on analysis time (+0.71 s), file size (+3.9 MB), and scanning time (+0.48 s).

Regarding the correlations between technical parameters, a very strong correlation was identified between file size and analysis time (ρ = +0.96, *p* < 0.001), as well as between analysis time and total tissue pixel count (ρ = 0.96), both statistically significant at *p* < 0.001. These results indicate that larger files with more tissue content are key determinants of computational processing time. Tissue coverage correlated strongly with analysis time (ρ = +0.732, *p* < 0.001) and moderately with file size (ρ = +0.667, *p* < 0.001). The analysis time showed a moderate correlation with the scanning time (ρ = +0.507, *p* < 0.001). The file size correlated weakly with the scanning time (ρ = +0.486, *p* < 0.001), as did the tissue coverage (ρ = +0.421, *p* < 0.001). The correlation between tissue coverage and the percentage of artifacts was positive but very weak (ρ = +0.121, *p* = 0.005). It should also be noted that a weak correlation was observed between total tissue pixel count and the percentage of artifacts (ρ = 0.22; *p* < 0.001), indicating that a larger tissue area has minimal association with the relative proportion of artifacts (Fig. 6A). All statistically significant results remained significant after Benjamini–Hochberg correction for multiple comparisons; no comparison or correlation changed its significance status following adjustment.

Multiple linear regression analysis was conducted to evaluate the predictive relationships between variables. The first model, which evaluated the technical predictors of the quantity of artifacts (dependent variable: total percentage of artifacts; independent variables: file size, analysis time, and scanning time), revealed a determination coefficient of *R*^2^ = 2.7%, indicating that the technical parameters explain a small proportion of the variance observed in the artifacts. The second model evaluated the impact of artifacts on analysis time (dependent variable: analysis time; independent variables: tissue coverage and total percentage of artifacts), obtaining *R*^2^ = 65.2%. The regression coefficient for the percentage of artifacts was β = +0.71 s (*p* < 0.001). The third model evaluated the impact of artifacts on scanning time (dependent variable: scanning time; independent variables: tissue coverage and total percentage of artifacts), obtaining *R*^2^ = 30.9%. The regression coefficient for the percentage of artifacts was β = +0.48 s (*p* = 0.026). The fourth model evaluated the impact of artifacts on file size (dependent variable: file size; independent variables: tissue coverage and total percentage of artifacts), obtaining *R*^2^ = 61.0%. The regression coefficient for the percentage of artifacts was β = +3.9 MB (*p* = 0.009; Fig. 6B).

## Discussion

### Operational performance and processing efficiency

This study demonstrates the feasibility and efficiency of implementing an open-source AI algorithm as an automated WSI quality assessment tool in a high-productivity digital pathology lab workflow context, highlighting its potential for integration into continuous quality control systems. The present study analyzed 544 WSI from biopsies processed in a single day, allowing a systematic evaluation of the scanning quality through the automated GrandQC+ system. The total scanning time was approximately 7 h and 10 min and the high success rate in slide digitization (100%) and the absence of technical failures reflect the robustness of the scanning process, the adequacy of the operational parameters adopted, but above all, the assessment and technical improvement of histological processing, its validation process, its components, and workflow. The volume of data generated (328 GB) is similar to that described in other studies, confirming the significant impact of digital archiving on the computational infrastructure of anatomical pathology departments.^4^, ^19^, ^35^, ^45^

The complete anonymization of WSIs, achieved in only 10 min, demonstrates and reinforces that this step can be effectively incorporated into a clinical research digitization process. It ensures compliance with ethical and regulatory standards without sacrificing operational efficiency. This is especially important under the GDPR, which sets strict rules for handling and storing biomedical data.^53^

GrandQC+’s analytical processing time took 8 h and 39 min, demonstrating the high computational load inherent in analyzing high-resolution histopathological images. The mean analysis time (57 s per WSI) reflects adequate computational performance, even under high processing load conditions. The median value, which is considerably lower at 24 s, indicates an asymmetric distribution where most cases are processed quickly, whereas larger WSIs require longer execution times. These results are in line with those of Weng et al. (2024), who indicate that the tool allows the analysis of individual slides in less than a minute.^45^ With regard to the time required for analysis/quality control by WSI, it should be noted that, despite the greater experience and technical knowledge of the histotechnologist, which should always be valued and taken into account, and however well-defined, the established quality control criteria may be, we consider it very difficult to perform manual quality control in a consistent, rapid, reproducible, and analytical manner, in the quantity produced during a working day, in the time achieved and demonstrated here by the algorithm. This is corroborated by Browning et al. (2024), who emphasize the need for this automation, noting that manual quality control is inefficient and prone to errors, requiring excessive human resources that many labs do not have.^22^, ^35^

### Artifact prevalence, distribution, and severity

From the perspective of image quality, the prevalence of artifacts was remarkably high, with 97.1% of the WSIs showing at least one detectable artifact, that is, at least one pixel is interpreted/evaluated as an artifact. Despite this high detection frequency, the average artifact load was 4.70% of the tissue area, with a median of only 1.46% (IQR: 0.39%–4.20%; 95% CI: 1.23%–1.73%) indicative of generally satisfactory digitization patterns. This discrepancy between prevalence and extent suggests that, although artifacts are ubiquitous, their magnitude is often minimal and clinically tolerable. In this case, the literature states that multiple artifacts are present in virtually all histological slides digitized by modern systems, confirming that most flaws are focal, and that the challenge is not only to detect the artifact, but to quantify its severity in order to decide whether the slide/WSI meets histological quality criteria compatible with its use for diagnosis or not.^18^, ^35^, ^45^, ^56^, ^57^

**Fig. 7:**
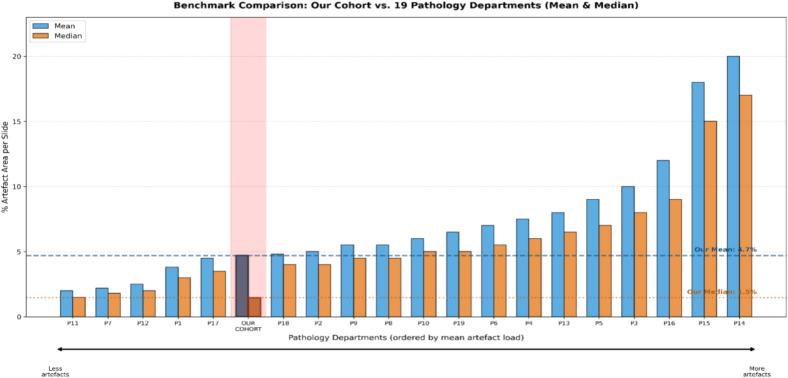
Benchmark comparison of mean and median artifact load (% area per slide) across 20 pathology departments. Our cohort (highlighted) ranked 6th/20, with values below the international average. Adapted from Weng et al. (2024).

The contextualization of the results obtained within the framework of the multi-center GrandQC validation study, published by Weng et al. (2024), allows for the assessment of the relative positioning of the present cohort against 19 reference pathology departments.^45^ Our results place it among the departments with the lowest proportion of tissue area affected by artifacts. Compared with the international benchmark, these results position our lab approximately 6th out of the 20 departments evaluated. The particularly low median (1.46%) suggests that the majority of WSIs exhibit high quality, with a minority of cases inflating the arithmetic mean. Our lab stands out for having the lowest median of all departments, matched with the lowest median lab in the study, indicating digitization and histological preparation standards consistent with international best practices in digital pathology. This means that, despite the average, most of our WSIs have excellent quality—it is the outlier cases with a high load of artifacts that pull the average up (Fig. 7).

A methodologically relevant finding emerged from the analysis of the pen markings class. Because the digitized sample consisted of original slides produced before diagnosis and before assignment to the pathologist, the ground truth for this class could be established with certainty: manual verification confirmed the absence of pen markings across all 544 analyzed WSIs. This confirmed-negative population allowed an exploratory, class-specific specificity estimate. Applying the threshold of clinically negligible involvement defined in this study (artifact-affected tissue below 1%), the algorithm correctly classified 486 of the 544 WSIs as free of relevant pen markings, corresponding to a specificity of 89.3% (95% CI, Clopper–Pearson, 86.4%–91.8%); when any detection was considered, regardless of magnitude, specificity was 16.5% (95% CI 13.5%–19.9%). Both figures are reported in the interest of transparency. The contrast between them is consistent with the very low magnitude of the detections, which affected on average 0.48% and a median of 0.06% of the tissue area—predominantly below the threshold of clinical relevance. As this analysis is restricted to a single artifact class and to a population composed solely of confirmed negatives, sensitivity, positive-predictive value, and κ are undefined; it should therefore be read as a partial, exploratory characterization rather than a formal accuracy validation. In some cases, the residual detections were observed to coincide with areas of tissue cauterization and with India ink applied for surgical margin delineation—chromatic and morphological features that the algorithm appears to interpret as ink markings. Given that the system does detect some form of image alteration, even where this does not correspond to true pen markings, the category was renamed other artifacts, acknowledging the detection of image alterations whose precise nature was not systematically characterized.^45^, ^58^

The analysis of the relative contribution of each type of artifact to the total affected area revealed a clear hierarchy. Dark spots emerge as the dominant artifact, occupying an average of 1.89% of the tissue area and representing 40.2% of the total artifact load. They were followed by out-of-focus artifacts (1.18%; 25.2%), folded tissue (0.84%; 17.9%), other artifacts (0.48%; 10.2%), and air/edge (0.30%; 6.5%).

### Operational implications for lab quality management

This distribution has relevant implications for the optimization of histological processing and scanning protocols, suggesting that interventions aimed at reducing dark spots and improving autofocus would have the greatest impact on the overall quality of the images. Most of these artifacts except folded tissue are often associated with coverslipping methodology, as an artifact of the mounting medium, dust, imperfections, or lack of quality of the coverslip glass. Testing alternative coverslipping method like film would be a measure to be taken into account in improving quality results.^59^

Beyond these preparation-related artifacts, a different kind of practical caveat concerns the other artifacts class. The specimen types most likely to exhibit this pattern can be inferred from the morphological features illustrated in Fig. 3, together with the documented composition of the sample: urological specimens obtained by endoscopic resection, such as transurethral resections of the prostate and bladder, and gastrointestinal specimens obtained by diathermy-loop polypectomy, are those whose cauterized tissue edges most plausibly account for the residual detections, although the irreversible anonymization of the WSIs precludes case-by-case confirmation. Labs implementing GrandQC—or comparable tools with a pen-marking detection class—whose case mix includes endoscopic or surgical specimens with cauterized margins should therefore verify this category critically before adopting it as a quality-control metric, given the possibility of systematic false-positive detections arising from the confusion between cauterization and ink.

It should be noted that the artifact categories detected by the tool are not univocally attributable to a single stage of the workflow. A given artifact type may arise from more than one origin: out-of-focus regions, for instance, may reflect scanner focusing limitations but also optical interference from the mounting medium, whereas dark spots may originate from debris on the scanner light path, on the coverslip, or on the section surface itself. Different classification schemes have been proposed in the literature—Kanwal et al. distinguish artifacts at the tissue, slide, and scanner levels, whereas Brixtel et al. separate them according to whether they are dependent on or independent of sample preparation—yet no single consensual taxonomy has been established, and artifacts remain, as noted by the latter authors, intrinsically challenging to define.^18^, ^56^ For this reason, each flagged case requires individual human review to determine the most probable origin and the appropriate corrective action, whether re-digitization or repetition of the histological technique. Beyond image quality itself, the multi-class nature of the artifact data carries operational value. Because specific artifact types map onto distinct stages of histological processing—tissue folds reflecting sectioning quality, air, and edge artifacts relating to coverslipping, and dark spots or pen marks pointing to slide preparation before scanning—their systematic and quantitative monitoring allows the root causes of recurring quality deviations to be identified and addressed. This shifts artifact detection from a passive, qualitative quality metric towards an active management tool, supporting the optimization of workflows and the reduction of costs associated with poor slide quality. Such benefits, however, are best realized through a structured, phased implementation rather than an immediate full deployment.

The predominance of dark spots and out-of-focus regions as the principal artifacts identified aligns with existing literature, which characterizes these defects as recurring outcomes of variations in focus and non-uniform illumination during the scanning process,^19^, ^35^, ^45^ and it is particularly dangerous because it can lead to “silent failures” in AI models, where the algorithm makes mistakes without warning.^23^

Based on our cohort distribution, four statistically grounded thresholds were identified for quality stratification: <1% designated as “perfect” quality, representing a clinically negligible artifact load, where less than 1% of tissue area is potentially compromised that can be interpreted for both human diagnosis and computational pipelines^23^; 5% corresponding to the 75th percentile; 7.5% aligned with international benchmark standards^45^; and 10% derived from Tukey's outlier detection method (Q3 + 1.5 × IQR = 9.91%). The latter was adopted as the primary threshold for distinguishing acceptable from severe quality, yielding an acceptable quality rate of 87.7%, while the <1% cut-off identified 42.5% of WSIs as achieving optimal digitization quality suitable for both diagnostic assessment and computational analysis pipelines, 35.8% good (>1%–≤5%) and 9.4% moderate (>5%–≤10%), whereas 12.3% were of high severity (≥10%), which are cases that require priority attention in quality monitoring systems and highlight the need for automatic, real-time artifact detection systems capable of flagging critical cases that may compromise diagnostic interpretation. It is noteworthy that, although automated artifact detection tools such as GrandQC+ provide quantitative area measurements, categorical severity stratification remains dependent upon the user and/or subjective expert consensus, without explicit quantitative thresholds, as previously described. Nevertheless, Hossain et al. demonstrated that severity-based classification achieves 97% correlation with expert assessment, supporting the clinical relevance of threshold-based quality stratification approaches. Building upon these principles, and in the absence of established consensus thresholds for artifact burden stratification in digital pathology, together with the quantitative measurements provided by the tool, our quality stratification framework derives thresholds not only empirically from cohort distribution statistics but also from internal expert consensus and validation, thereby providing reproducible, data-driven quality categories that converge the strengths of both approaches.^45^, ^55^ Comparative analyses between acceptable and severe quality groups demonstrated consistent results across all proposed thresholds, with no statistically significant differences observed in technical parameters regardless of the cut-off employed, thereby corroborating the robustness of this classification framework as a data-driven criteria for quality stratification in routine digital pathology practice. The severity stratification adopted in this study should be interpreted as an exploratory, institution-specific framework rather than a definitive classification. The cut-offs emerged from the artifact-load distribution of a single cohort and reflect the digitization and histological standards of one lab; their value lies in enabling structured internal quality monitoring, not in providing a transferable diagnostic standard, and their external validity remains to be established. Their adoption in other settings would require adaptation to local case mix and digitization conditions, and ideally prospective validation against diagnostic or computational performance.

Notably, only one case required a rescan due to a “stitching” error, thus highlighting the effectiveness of the quality control process and the reduced operational impact of automatically detected technical anomalies. The histotechnologist only needed to manually review the WSIs considered severe (*n* = 67) automatically by the algorithm, which constitutes just over 10% of the biopsy WSIs produced on that day, making this process more cost-effective by limiting manual quality control only to cases that really needed to be reviewed. In the study by Ferreira et al. 2023, the repetition rate—here defined as the proportion of WSIs requiring re-digitization (rescan), equivalent to the rescan rate referred to above—was 0.6% during the digital pathology validation period, which would correspond to approximately three repetitions in this cohort. In this sense, there was an improvement in quality standards and repetition rates between the two studies.^34^

Correlational analysis revealed consistent patterns in the associations between technical parameters and the occurrence of artifacts. The most robust correlation identified was between the file size and the analysis time (ρ = 0.963; *p* < 0.001), indicating an almost deterministic relationship between these variables. In practical terms, this means that larger files require proportionally more processing time. Similarly, the correlation between the percentage of tissue and the analysis time (ρ = 0.732; *p* < 0.001) confirms that images with a larger tissue area require greater computational effort. Contrasting with these strong associations, the correlation between the percentage of artifacts and the analysis time proved to be only weak (ρ = 0.25; *p* < 0.001). This finding is particularly relevant, as it indicates that the presence of artifacts does not constitute a significant limiting factor for the performance of the GrandQC+ system, reinforcing its usefulness for implementation in continuous monitoring workflows. The correlation between tissue area and the percentage of artifacts was also weak, although statistically significant (ρ = 0.12; *p* = 0.005). This result explains minimal variation and suggests that tissue extension is not a primary determinant of the amount of clinically relevant artifacts. This is an important finding for longitudinal monitoring of digital quality, indicating that an increase in tissue area does not substantially compromise image quality, although it has to be taken into account. These data corroborate the idea that scanner performance remains relatively stable regardless of the morphological complexity of the samples and their size, thus increasing confidence in the applicability of GrandQC+ in various lab environments.

Multiple linear regression models provided an accurate quantification of the relationships between variables, allowing for the estimation of the independent impact of artifacts on performance parameters. The first model, which evaluated the technical predictors of the quantity of artifacts, revealed a determination coefficient of only *R*^2^ = 2.7%. This remarkably low value unequivocally demonstrates that the technical parameters of digitization (file size, analysis, and scanning times) explain less than 3% of the variability observed in the occurrence of artifacts. The direct implication is that the origin of artifacts depends predominantly on pre-analytical factors—particularly the quality of histological preparation, presumably from fixation, processing, sectioning, and staining—rather than variables inherent to the digitization process.^1^, ^18^, ^19^, ^23^, ^31^, ^32^, ^33^, ^60^ Other models quantified the impact of artifacts on performance metrics: Analysis time (*R*^2^ = 65.2%)—each 1% increase in the area of artifacts was associated with a 0.71-s increase in processing time (*p* < 0.001). Although statistically significant, this impact is operationally modest; scanning time (*R*^2^ = 30.9%)—the effect of artifacts was even more attenuated, with each additional 1% contributing only 0.48 s (*p* = 0.026); file size (*R*^2^ = 61.0%)—each 1% of artifacts added approximately 3.9 MB to the file size (*p* = 0.009), reflecting the additional information needed to encode artifact regions. Thus, multivariate regression demonstrates that artifacts cause some measurable influence on performance parameters, however, their impact is quantitatively modest and does not compromise the operational viability of the automated quality control system.

### Limitations and future directions

The interpretation of the present findings warrants consideration of several methodological aspects as study limitations. The cohort analyzed corresponds to the complete and consecutive biopsy output of a single production day rather than a selected sample, which confers ecological validity by reflecting the true workload and case-mix of a high-throughput service without selection bias. Its retrospective, cross-sectional design, however, does not permit assessment of temporal variability in artifact rates, which may fluctuate with seasonal workload, equipment maintenance cycles, and operator-dependent factors. The magnitude of such variability is not negligible: in the original GrandQC validation, per-slide artifact content varied by up to 20% among slides from a single department. A single-day snapshot should therefore be complemented by longitudinal monitoring—a use case for which the GrandQC framework was explicitly designed, and a natural extension of the present work. Three potential sources of bias follow from this design: temporal selection bias, as the chosen day may not represent typical conditions—mitigated, though not eliminated, by its random selection; case-mix bias, because the day's distribution of specimen types may diverge from the annual distribution and artifact profiles are tissue-dependent; and the fact that the reported prevalences reflect the output of the GrandQC+ pipeline rather than blinded expert annotation.

Relatedly, the analysis did not stratify artifact burden by tissue type, precluding the identification of tissue-specific artifact profiles—for instance, in stereotactic breast or bone marrow biopsies, which require differentiated processing and pose greater architectural quality-assurance challenges. Such stratification would inform targeted pre-analytical interventions and refine quality benchmarks for specimen-specific workflows. A comparison between incisional and excisional biopsies, or with resection specimens, was likewise not feasible: the procedure subtype is not encoded in the image or recorded variables, and the irreversible anonymization of the WSIs removed the examination identifier needed to reconsult the LIS. The tissue origin of the specimens, by contrast, was preserved and is reported. The systematic characterization of resection specimens, for which GrandQC has been validated in its original multi-institutional study, multi-specimen study remains a defined direction for future work.

The single-center design, while ensuring procedural homogeneity, limits external validity: labs using distinct scanning platforms, processing protocols, or mounting techniques may exhibit divergent artifact profiles. All 544 WSIs were digitized on a single Aperio GT450DX scanner—a deliberate choice that allowed scanning time to be attributed unambiguously and reflects routine practice, in which one scanner is dedicated to biopsies and urgent cases. Inter-scanner variability, a documented capability of the GrandQC benchmark, was consequently not assessed and represents a defined avenue for future work, well supported by the lab's three-scanner infrastructure. Moreover, GrandQC+ quantifies morphological artifacts detectable at the image level, but does not capture upstream quality deviations preceding digitization—namely inadequate fixation, section thickness variability, or staining quality. The objective, quantitative assessment of staining quality in particular falls outside the scope of artifact detection and constitutes a complementary direction for future work, towards a more complete quality-control framework.

A final consideration concerns the deployment model. The analysis was conducted retrospectively, as a single overnight batch on a local workstation without integration into the LIS, consistent with the nature of the study as an operational feasibility evaluation of a beta-stage adaptation of GrandQC, aimed at establishing proof of concept rather than production deployment. The viability demonstrated here provides the basis for a subsequent prospective study integrating the GrandQC+ pipeline into the LIS, enabling artifact-based quality control in real time, concurrently with routine digitization. In such a setting, analysis would be triggered automatically by an HL7 message issued upon completion of digitization—the same infrastructure already linking the scanners to the information system—feeding a dedicated quality-control database. Two complementary outputs would support the workflow: a per-WSI quality flag returned to the LIS, and a review queue restricting manual inspection to cases automatically classified as severe. Accumulated over time, these records would provide objective indicators for temporal monitoring, early detection of process or scanner deviations, and compliance with certification and accreditation requirements.

**Fig. 8:**
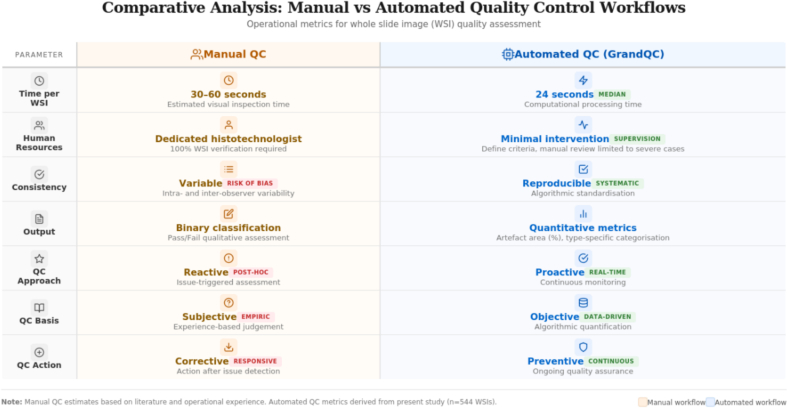
Comparative demonstrative scheme between manual versus assisted AI WSI quality control methodologies.

In summary, these findings support the incorporation of GrandQC+ as an automated element of continuous quality control in digital pathology workflows. Automated quality control using GrandQC+ offers significant operational advantages over manual QC workflows, including reduced processing time, decreased reliance on dedicated personnel, elimination of observer-dependent variability, and generation of quantitative rather than binary quality metrics. Most fundamentally, automated QC enables a paradigm shift from reactive, post-hoc corrective interventions towards proactive, real-time, data-driven continuous quality assurance—a transition essential for scalable digital pathology implementation and reliable AI-assisted diagnostics (Fig. 8). The methodology demonstrates robustness, scalability, and compliance with data inter-operability and traceability standards, which are crucial for the certification and accreditation of digital diagnostic systems.

## Conclusion

The transition to digital pathology represents a fundamental advance in modern pathology labs, promising gains in efficiency, automation, security, remote collaboration capabilities, and the integration of AI tools to support diagnosis—computational pathology. However, whereas this technology offers enormous advantages, it also introduces unique quality assurance challenges, namely the prevalence of artifacts in digitized histological slides (WSIs). Managing these artifacts is not only an operational task but also a strategic imperative to ensure diagnostic reliability, workflow efficiency, and the future success of integrating AI into clinical practice.

The results obtained indicate that the implementation of GrandQC+ represents a technically sound and operationally feasible methodology for continuous quality control in digital pathology. This approach facilitates the systematic monitoring of substantial volumes of WSIs without adversely affecting lab workflows. The integration of processing times that are compatible with clinical practice, a low technical failure rate, and comprehensive artifact detection capabilities positions this tool as a critical element in the establishment of scalable digital diagnostic infrastructures that adhere to regulatory and accreditation standards. Ideally, and contrary to what has been tested here, quality control should not be an exercise automatically performed in batch overnight, but rather, with this tool properly integrated with the computer system and/or LIS, it should be performed individually and constantly as each WSI is produced and before being distributed to the respective pathologist if it meets the quality conditions for this, constantly feeding a database that will contribute to continuous lab improvement and its quality management. This is a first step that will constitute a future approach to be carried out/implemented in our institution. In this context, future perspectives include prospective continuous monitoring approach would provide a more robust understanding of daily and weekly quality fluctuations, enabling trend analysis and early identification of systematic deviations, including under which conditions such deviations occur and their potential predictive value. Additionally, the findings of the present study underscore the relevance of investigating in the future the correlation between pre-analytical factors, such as cold ischemia time, fixation duration, tissue processing, section thickness, staining intensity, and coverslipping methodology, as well as tissue type/origin, with the quantity of detected artifacts and severity as a WSI quality metric. Through the analysis and longitudinal monitoring of such data, predictive tools could be developed to estimate the probability of artifact occurrence under specific conditions, thereby enabling the implementation of directed pre-analytical corrective measures and the calibration of quality thresholds tailored to distinct specimen categories. Moreover, these data hold potential utility for quality management purposes, constituting indicators and critical variables within risk management frameworks, quality assurance and the continuous improvement of diagnostic practice.

The pronounced correlation between analysis time, file size, and tissue quantity confirms that the complexity and informational content of the images are the primary determinants of computational effort. This provides a quantitative basis for hardware capacity planning and for the prospective architectural optimization of quality algorithms. Concurrently, the weak correlation observed between tissue extent and the percentage of artifacts, along with the modest influence of artifact load on processing time, underscores the notion that GrandQC+ is adept for heterogeneous contexts, maintaining consistent performance despite morphological variability and differences in preparation quality. From a quality assurance perspective, the identification of a minor yet clinically significant fraction of WSIs exhibiting a high artifact load highlights the added value of automated and continuous monitoring. This capacity supports objective decision-making regarding the necessity for repeat scanning, the exclusion of low-quality cases, or the requirement for corrective adjustments in scanning or pre-analytical parameters/factors, respectively. These findings advocate for a “quality by design” framework, wherein the objective assessment of image integrity and legibility is established as a systematic prerequisite before human or algorithmic analysis. This approach contributes to the reduction of variability, mitigates the risk of diagnostic error, and enhances the reliability of AI pipelines in the field of pathology.

The adoption of AI algorithms for automated quantitative quality control in digital pathology represents a structural advancement over traditional manual evaluation models, as it allows for objective, reproducible, and scalable measurements of image quality, mitigating inter- and intra-observer variability, reducing dependence on subjective judgements, and overcoming the limitations inherent in time- and human resource-intensive manual procedures.

The present study provides evidence to support the integration of adapted GrandQC+ as a structural module within digital quality management systems in pathology, consistent with contemporary hospital digital transformation strategies and the growing demand for reproducibility and transparency in the large-scale application of histopathological data.

In summary, the evidence presented supports that implementing adapted GrandQC+ is an investment in the foundational integrity of our entire digital pathology operation. It will allow us to move from reactive quality control to a proactive system of continuous, data-driven quality assurance, yielding transformative improvements in diagnostic accuracy, operational intelligence, and process efficiency.

## Declaration of competing interest

The authors declare that they have no known competing financial interests or personal relationships that could have appeared to influence the work reported in this article.
